# Effects of hyperoxia in pediatric perfusion patients: a scoping review

**DOI:** 10.1051/ject/2025048

**Published:** 2025-12-17

**Authors:** Mariah DeRenzo, Vanessa Velazquez-Milton, Stepney Johnson

**Affiliations:** 1 Emory University Perfusion Program Atlanta GA 30322 USA

**Keywords:** Cardiopulmonary bypass (CPB), Hyperoxia, Cyanotic, Acyanotic, Pediatric, Perfusion

## Abstract

Hyperoxia in pediatric cardiopulmonary bypass patients is associated with adverse effects, including reoxygenation injury, increased mortality, heightened inflammatory response, and cerebral injury. Although prior research has demonstrated a correlation between these adverse effects and hyperoxia during cardiac surgery, definitions of “hyperoxia’ vary across the literature. This scoping review synthesizes findings from PubMed, CINAHL, Embase, and Web of Science to categorize the adverse effects of hyperoxia based on age range, presence of cyanosis, and PaO_2_ levels. The results highlight the need for standardized oxygenation management protocols in pediatric bypass procedures and a consistent definition of “hyperoxia” for cyanotic and acyanotic patients.

## Introduction

Historically, hyperoxia has had a reputation for its therapeutic advantages surrounding cardiac surgery. These included preconditioning the myocardium to make ischemia more tolerable, reducing postoperative wound infection rates, and diminishing gaseous microemboli in the cardiopulmonary bypass circuit [1]. However, more recent research surrounding the effects of hyperoxia reveals the negative impact it may have on the pediatric population. In general, hyperoxia associated with cardiopulmonary bypass can result in lung injury, increased systemic reactive oxygen species generation, and exacerbation of reactive oxygen species-mediated myocardial injury at the time of reperfusion [2]. One study found that hyperoxia during cardiopulmonary bypass was associated with four-fold greater odds of mortality in infants within 30 days after surgery [3]. The study stated that validating their data among other age populations is necessary to understand the full effects of hyperoxia on cardiopulmonary bypass patients [3]. The initial review of this study sparked interest in a further scoping review of other available studies. The lack of consistent definitions for hyperoxia and the overall lack of studies on the adverse effects of hyperoxia became evident.

The topic for this scoping review was selected based on the variability of oxygenation across the pediatric perfusion practice. Current data were organized based on the level of hyperoxia with the goal of providing a recommended oxygenation strategy to limit the adverse effects. The purpose of this review was to provide a concise manuscript with several relevant hyperoxia studies for pediatric cardiac surgery. The following question was asked: In pediatric cardiac surgery with cardiopulmonary bypass, what are the adverse effects of hyperoxia compared to normoxia? There is literature surrounding the effects of hyperoxia in pediatric bypass patients, ranging from neonates to adolescents. However, there is a lack of a consistent definition of hyperoxia. This scoping review aims to provide a recommendation regarding oxygenation management during bypass for pediatric perfusionists based on the summarized adverse effects seen in hyperoxia. This review also aims to identify gaps in the literature and synthesize a new outlook on hyperoxia through the arrangement of its results.

## Methods

The authors determined that a scoping review was the most appropriate choice for discussing this topic, considering the gaps in literature and the lack of oxygen care standardization. Thirteen studies were selected for data analysis, which included eight randomized trials and five retrospective studies. The studies took place in many centers around the world, including the United States, Japan, the United Kingdom, China, Italy, and Turkey.

**Figure 1:**
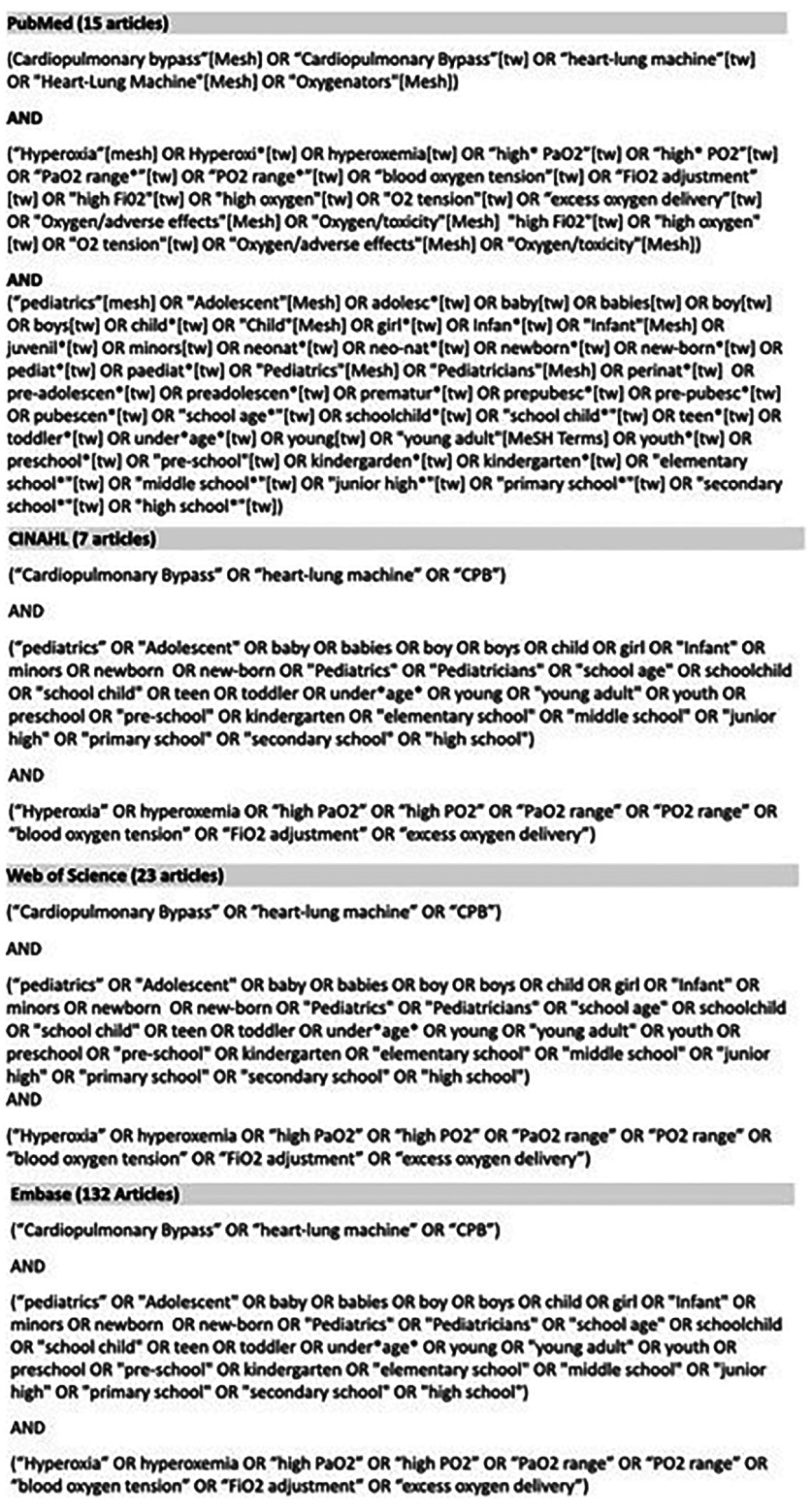
Search terms used for literature search.

**Figure 2:**
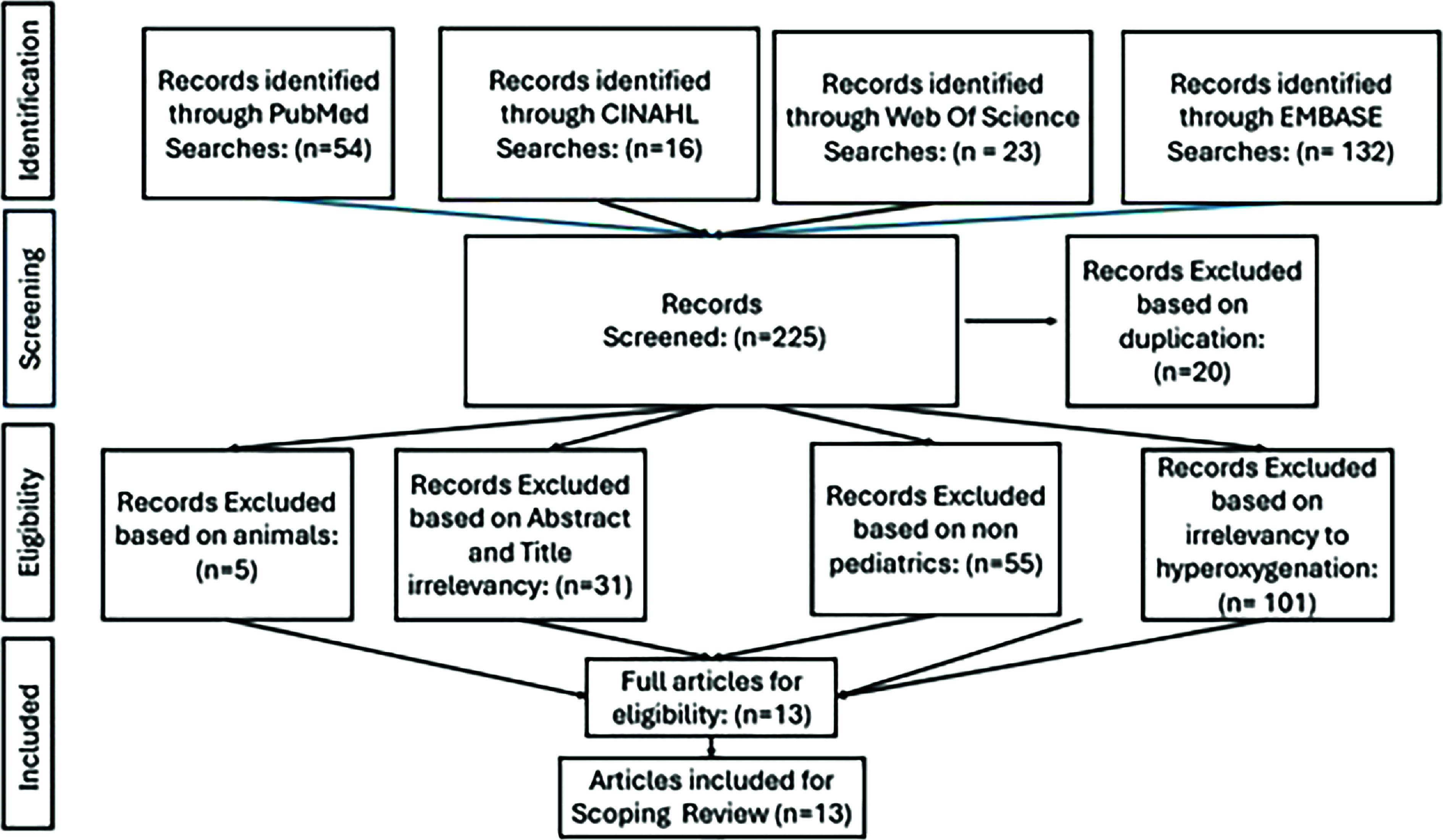
PRISMA diagram for article selection.

This scoping review encompassed data collection from multiple databases, including Embase, Web of Science, PubMed, and CINAHL, with the majority of the information coming from PubMed and CINAHL. Detailed search terms related to cardiopulmonary bypass (CPB), adverse effects, and pediatrics were utilized. Similar word choices and alternative variations were also chosen to provide a broader search. Figure 1 outlines the complete list of search terms. The collection included all data provided for pediatric patients aged from newborn to 18 years who were on CPB and exposed to hyperoxia. The results from the search terms and exclusion/inclusion criteria are highlighted in the PRISMA diagram in Figure 2. Exclusion criteria consisted of animal studies, studies that refer only to extracorporeal membrane oxygenation (ECMO), studies done on adult patients, and studies that were not related to hyperoxygenation. For these purposes, a *p*-value of <0.05 was considered statistically significant for all articles.

Duplicate articles were noted from other databases such as Embase, Web of Science, PubMed, and CINAHL. Web of Science and Embase provided additional articles, but most were excluded due to this review’s exclusion criteria. Covidence helped narrow the database usage to CINAHL and PubMed due to repetitions.

By appraising the literature and summarizing the findings, this review outlined the adverse effects seen and organized the information in one place to spread awareness of this issue. Figures and tables were utilized for data management to compare results and analyze them.

Findings were organized based on age range, cyanosis (cyanotic or acyanotic), adverse outcomes, and PaO_2_ ranges. Patterns and positive correlations between certain variables became apparent, such as cyanosis and reperfusion injury. Microsoft Excel spreadsheets were created to document and organize findings and are included in Results section. During this research, it was discovered that definitions of hyperoxia vary between publications. To mitigate this problem, adjustments were made to the proposal to reflect the effects of hyperoxygenation on bypass and include the range of various definitions between articles.

## Results

All studies were compared, filtering for specific characteristics, including source, sample size, age, cyanotic/acyanotic, definition of hyperoxia, and outcomes. The characteristics were organized to easily compare data points (see Table 1). Through this method of result analysis, there was a visible connection between cyanotic/acyanotic patients and specific outcomes, as shown in Tables 2 and 3.

**Table 1:** Article overviews with notable characteristics of each. Definitions of hyperoxia are mentioned to highlight the variety. RI: reoxygenation injury; ROS: reactive oxygen species; IR: inflammatory response; LOS: length of stay.

Source	Sample size	Age range	CPB duration mean (min)	Definition of Hyperoxia	Cyanotic?	Outcome
Allen et al. [4], US	21	Infants	Not mentioned	PaO_2_ of 400–550 mmHg	Cyanotic	RI, ROS
Beshish et al. [3], US	469	<1 year	128 min cyanotic/98 acyanotic	PaO_2_ of >313 mmHg	Not mentioned	Mortality, including LOS
Bulutcu et al. [5], Turkey	24	Infants	116	PaO_2_ of 300–350 mmHg	14 cyanotic/10 acyanotic	RI ROS
Caputo et al. [6], UK	64	6 months–4 years	86	PaO_2_ of 150–180 mmHg in cyanotic patient	Cyanotic	RI, cerebral injury, hepatic injury
Caputo et al. [7], UK	169	4 months–4 years	96	PaO_2_ of 150–200 mmHg in cyanotic patient	Cyanotic	Inc transfusion and kidney injury
Cashen et al. [8], US	484	<19 years	Not mentioned	PaO_2_ of >200 mmHg on ECMO	n/a	Mortality, kidney failure, inc LOC
Gretchen et al. [9], US	100	>1 month–18 years	73	PaO_2_ of 150–180 mmHg	Mostly acyanotic	Hemolysis, ROS
Kagawa et al. [10], Japan	22	2–21 years	122	PaO_2_ of 200–300 mmHg	Acyanotic	IR, inc lung injury, ROS
Modi et al. [11], UK	29	4.5 months–14.5 years	Not mentioned	No definition	20 cyanotic/9 acyanotic	RI
Sznycer-Taub et al. [12], US	93	5–20 days	156	PaO_2_ of >193 mmHg on ECMO	n/a	Mortality, kidney injury, inc LOC
Varrica et al. [13], Italy	48	Infants	81	No definition	Cyanotic	RI, cerebral injury
Yang et al. [14], China	376	6 months–6 years	123	PaO_2_ of >250 mmHg	Cyanotic	RI
Zhu et al. [15]	20	Pediatric	Not mentioned	CPB initiation with FiO_2_	Cyanotic	RI, cerebral injury

**Table 2:** Adverse effects noted for cyanotic-only patients. Age ranges are also noted. RI: reoxygenation injury; ROS: reactive oxygen species; IR: inflammatory response; LOS: length of stay.

Source	Cyanotic?	RI	Mortality	Kidney injury	ROS	IR	Inc LOS	Hepatic Injury	Hemolysis	Cerebral injury	Inc Transfusion	Inc Lung injury	Age range
Allen et al. [4]	Yes	X			X								Infants
Caputo et al. [6]	Yes	X						X		X			6 months–4 years
Caputo et al. [7]	Yes			X							X		4 months–4 years
Varrica et al. [13]	Yes	X								X			Infants
Yang et al. [14]	Yes	X											6 months–6 years
Zhu et al. [15]	Yes	X								X			Pediatric

**Table 3:** Adverse effects noted for acyanotic and mixed articles. Age ranges are also noted. RI: reoxygenation injury; ROS: reactive oxygen species; IR: inflammatory response; LOS: length of stay.

Source	Cyanotic?	RI	Mortality	Kidney injury	ROS	IR	Inc LOS	Hepatic Injury	Hemolysis	Cerebral injury	Inc Transfusion	Inc Lung injury	Age range
Kagawa et al. [10]	No				X	X						X	2–12 months
Cashen et al. [8]	n/a		X	X									<19 years
Beshish et al. [3]	n/a		X				X						<1 year
Sznycer-Taub et al. [12]	n/a		X	X			X						5–20 days
Modi et al. [11]	Both	X											4.5 months–14.5 years
Gretchen et al. [9]	Both								X				>1 month–18 years
Bulutcu et al. [5]	Both	X											Infants

Cyanotic patients [4, 6, 7, 13–15] consisted mostly of the younger age group, including infants and children up to 6 years of age. Reoxygenation injury was the primary adverse effect seen in cyanotic patients, followed by cerebral injury (Table 2). The acyanotic/mixed table [3, 5, 8–12] (see Table 3) also includes studies where cyanosis was not mentioned. The most common adverse effect seen in this patient population was an increased mortality rate and reactive oxygen species, followed by reoxygenation injury and kidney injury. Another pattern identified included relationships between outcomes and the age range of patients. In the younger age groups, there is a more frequent prevalence of reoxygenation injury and cerebral injury. As the data suggest, this may be due to the higher prevalence of cyanosis in that population.

The wide range of definitions for hyperoxia was a challenge encountered throughout the entirety of this research. Consequently, this is the rationale for the inclusion of each article’s specific parameters in this scoping review. The definitions of hyperoxia ranged from a PaO_2_ of 150 to 550 mmHg during bypass. Another article defined hyperoxia as a starting FiO_2_ of 100% on bypass and then lowering FiO_2_ following aortic cross-clamp placement compared to starting FiO_2_ at 21% [15]. The PaO_2_ levels associated with 100% FiO_2_ were between 350 and 400 mmHg. There were a variety of adverse outcomes, the most common being reoxygenation injury [4–6, 11, 13–15]. Kidney injury, cerebral injury, reactive oxygen species, hepatic injury, increased inflammatory response, hemolysis, lung injury, increased length of stay, and increased need for transfusion were also observed [3, 6–10, 12, 13, 15]. These outcomes are summarized in Table 4.

**Table 4:** Adverse effects noted for all patients in the thirteen articles. RI: reoxygenation injury; ROS: reactive oxygen species; IR: inflammatory response; LOS: length of stay.

Source	Cyanotic?	RI	Mortality	Kidney injury	ROS	IR	Inc LOS	Hepatic Injury	Hemolysis	Cerebral injury	Inc Transfusion	Inc Lung injury
Allen et al. [4]	Yes	X			X							
Beshish et al. [3]	n/a		X				X					
Bulutcu et al. [5]	Both	X			X							
Caputo et al. [6]	Yes	X						X		X		
Caputo et al. [7]	Yes			X							X	
Cashen et al. [8]	n/a		X	X								
Gretchen et al. [9]	Both				X							
Kagawa et al. [10]	No				X	X						X
Modi et al. [11]	Both	X										
Sznycer-Taub et al. [12]	n/a		X	X			X					
Varrica et al. [13]	Yes	X								X		
Yang et al. [14]	Yes	X										
Zhu et al. [15]	Yes	X								X		

It is of note that the two Caputo articles did not study the same adverse effects, since they previously found that hyperoxia caused reoxygenation injury, hepatic injury, and cerebral injury [6, 7]. Also, Cashen et al. worked under the assumption that hyperoxia caused reoxygenation injury, ROS, and cerebral injury [8].

## Discussion

This scoping review shows the different ways in which pediatric cardiopulmonary bypass patients are susceptible to hyperoxic injury. Specifically, the data suggest there may be a positive correlation between hyperoxia and reoxygenation injury in the pediatric cyanotic patient population. The most common age range of this cyanotic group observed was neonates to 6-year-old children. Cyanotic patients have a history of hypoxic blood levels, and the extreme change in oxygen saturation upon initiation of cardiopulmonary bypass has led to an increased rate of reperfusion injury and cerebral injury in this patient population [5, 15, 16]. Zhu et al.’s study in particular suggested that during initiation of CPB, FiO_2_ should be set at a lower concentration (21%) to mimic the cyanotic patient’s natural oxygen level. FiO_2_ should slowly be increased to 30% and then 60% within the first ten minutes of bypass [15]. Interestingly, the data suggest that acyanotic/mixed patients experienced increased mortality as the most frequent adverse effect, while the cyanotic group had no presence of mortality associated with hyperoxia. There is difficulty in comparing cyanotic with acyanotic patient outcomes because of multiple variables. Cyanotic patients are typically younger during cardiopulmonary bypass because cyanosis requires earlier treatment. Acyanotic patients could go undiagnosed with their abnormality due to undetected symptoms. The results showing that mortality was not an adverse effect for the cyanotic group could be due to the young age of the patients, unrelated to hyperoxia. The length of bypass time can also differ between groups, as the procedures are often different for cyanotic and acyanotic patients. Some procedures can be seen in both groups, especially when multiple defects are corrected in a single procedure.

The adverse effects of hyperoxia are an overlooked aspect of patient safety during pediatric cardiopulmonary bypass. These findings suggest that hyperoxia is linked to significant adverse effects, highlighting the need for a more precise oxygen range in pediatric perfusion practices. Before making such advancements, it is essential to clearly define hyperoxia in the context of cardiopulmonary bypass. This research reveals a wide variability in how hyperoxia is defined, which not only complicates this scoping review but also hinders progress in improving this treatment approach. In addition, it was also difficult to distinguish if these adverse effects were strictly associated with hyperoxia, or if other perioperative factors and patient comorbidities contributed to their development. However, even with these difficulties, this scoping review was able to distinguish some associations within the results that will open the door for further research on this topic.

It is reasonable to assume that the duration of hyperoxia impacts the appearance of adverse effects. Total CPB times were documented in nine out of thirteen of the studies; however, it is not noted when hyperoxia occurs. The thirteen studies present another challenge when comparing them. The adverse effects were not clearly defined in any of the articles except for one. “Acute hypoxia followed by any abrupt reoxygenation results in an injury characterized by a decrease in systolic contractility, an increase in diastolic stiffness, and elevated pulmonary vascular resistance. This injury, which has been referred to as the reoxygenation injury, is mediated by oxygen free radicals” [4]. Even articles that had the same adverse effects used different biological markers to measure them. For example, serum troponin level, malondialdehyde (MDA), tumor necrosis factor α (TNF-α), and even reactive oxygen species (ROS) were all noted as markers for reoxygenation injury. ROS is its own adverse effect, but it can cause reoxygenation injury. This caused the article comparison to be inconsistent since ROS is not the only cause of RI.

Although an optimal oxygen delivery range to decrease the adverse effects of hyperoxia could not be established, this scoping review will bring awareness to the lack of standardized oxygenation management. If defined correctly, it could be more accessible to associate adverse effects with hyperoxia and begin to recognize patterns in the recovery outcomes of pediatric cardiac surgery patients.

## Implications for Perfusion Practice

This study highlights a connection between hyperoxygenation and adverse effects, with the most concerning outcomes including reoxygenation injury, cerebral injury, and increased mortality. The primary aim of this review is to raise awareness of these risks and recommend reducing oxygen levels when possible. Specifically, pediatric perfusionists can lower FiO_2_ and PaO_2_ during bypass initiation. While this review does not show a conclusive correlation, further research will likely improve on this subject. Both cyanotic and acyanotic patients demonstrated susceptibility to hyperoxia-related complications, emphasizing the need to limit PaO_2_ to a defined target range. This study also identified critical gaps in existing literature, such as the absence of a clear definition for hyperoxia, various pediatric age ranges, and the exclusion of cyanotic patients in prior research. Given the relationship between hyperoxia and adverse outcomes in this patient population, it is crucial for future studies to address these gaps with greater specification. The next step is to formulate a universal definition for hyperoxia in cyanotic and acyanotic patients undergoing cardiopulmonary bypass.

## Limitations

There is a significant gap in the literature on this topic, even though it is clearly associated with adverse effects in pediatric patients. The scarcity of relevant articles was so prominent that studies dating back to 1997 needed to be included. The main limitation is that some articles do not specifically define the parameters surrounding hyperoxia. The articles that do include definitions have differing ranges. This creates difficulty when attempting to standardize the results. Other articles do not specify what age group they are gathering data on and only mention that they are “pediatric” or “infant”. For example, an article uses the term pediatric to describe patients under 19 years old [8]. Thus, the pediatric age group was reviewed as a whole, rather than further narrowing the results. Another research challenge was determining if adverse outcomes are explicitly caused by hyperoxygenation or other intraoperative/patient-specific conditions, such as age, longer cardiopulmonary bypass times, and ventilation times. For this reason, this review focused on the significant outcomes directly attributed to hyperoxygenation. Furthermore, since some of the adverse effects of oxygen toxicity can be seen later in life, it is difficult to see the full spectrum of impact beyond the hospital stay. The lack of definitions for the adverse effects tested and differing biomarkers made it difficult to assess commonalities. Also, instances of adverse effects causing each other further complicate the findings. For example, ROS is an adverse effect, however, it can lead to reoxygenation injury as well. Additionally, some articles worked under the assumption that hyperoxia causes certain adverse effects, so they did not include them in their results. Cashen et al. noted previous understanding of hyperoxia’s association with ROS, RI, and cerebral injury [8]. Caputo had two articles that were reviewed, and they did not study the same adverse effects that they noted in the first article [6, 7]. The thirteen articles did not all test for the same adverse effects. It is possible that if the same biomarkers were looked at across all articles, the adverse effects could have been seen in other articles. Future research should assess all of the possible associated outcomes listed.

## Summary

In conclusion, this scoping review highlights the variability in definitions of hyperoxia and the associated adverse outcomes in pediatric cardiopulmonary bypass. By analyzing data from 13 studies, this review identified patterns linking cyanotic and acyanotic patient populations to specific outcomes, such as reoxygenation injury, cerebral injury, and increased mortality rates. These findings emphasize the need for standardized definitions and oxygenation management strategies for the pediatric population. Future research should focus on developing a universal definition of hyperoxia in this population and developing protocols to mitigate its adverse effects. Subsequent studies should explicitly state their definition of hyperoxia with PaO_2_ and FiO_2_ ranges, include all of the adverse effects seen in this review, along with any additional ones, define what biomarkers they are using to assess the adverse effects, state the age range, and include bypass durations. Additionally, while animal studies were excluded from this review, experimentation with animals has the potential to make breakthroughs if they follow the same guidelines listed above.

## Data Availability

The research data associated with this article are included within the article.
